# Stop-and-Go Mode: Sensor Manipulation as Essential as Sensor Development in Terrestrial Laser Scanning

**DOI:** 10.3390/s130708140

**Published:** 2013-06-25

**Authors:** Yi Lin, Juha Hyyppä, Antero Kukko

**Affiliations:** 1 Institute of Remote Sensing and Geographical Information Systems, Beijing Key Lab of Spatial Information Integration and Its Applications, Peking University, Beijing 100871, China; 2 Department of Remote Sensing and Photogrammetry, Finnish Geodetic Institute, 02430 Masala, Finland; E-Mails: juha.hyyppa@fgi.fi (J.H.); antero.kukko@fgi.fi (A.K.)

**Keywords:** stop-and-go, static terrestrial laser scanning, mobile terrestrial laser scanning, sensor manipulation

## Abstract

This study was dedicated to illustrating the significance of sensor manipulation in the case of terrestrial laser scanning, which is a field now in quick development. In fact, this quickness was mainly rooted in the emergence of new sensors with better performance, while the implications of sensor manipulation have not been fully recognized by the whole community. For this technical gap, the stop-and-go mapping mode can be reckoned as one of the potential solution plans. Stop-and-go was first proposed to handle the low efficiency of traditional static terrestrial laser scanning, and then, it was re-emphasized to improve the stability of sample collections for the state-of-the-art technology of mobile laser scanning. This work reviewed the previous efforts of trying the stop-and-go mode for improving the performance of static and mobile terrestrial laser scanning and generalized their principles respectively. This work also analyzed its advantages compared to the fully-static and fully-kinematic terrestrial laser scanning, and suggested the plans with more automatic measures for raising the efficacy of terrestrial laser scanning. Overall, this literature review indicated that the stop-and-go mapping mode as a case with generic sense can verify the presumption of sensor manipulation as essential as sensor development.

## 1. Introduction

With the vigorous emergence of various laser scanners, the technique of light detection and ranging (LiDAR) has undergone developments and applications in an explosive fashion. As one of its important branches, terrestrial laser scanning has also evolved quickly. This evolution was driven by the relevant demands in an extensive sense, *i.e.*, from fine-scale 3D object reconstruction [1] to sample acquisition for training large-scale parameter retrievals [2]. During this progress phase, it has also been gradually recognized that the traditional static terrestrial laser scanning (conventionally termed as TLS) [3] tends to suffer from low surveying efficiency. That is, as TLS scanners are often mounted onto tripods and their data-georeferencing operations are generally based on the location-indicating marks, moving such supporting bases and then re-placing the reference marks at each new position require a lot of labors. This somehow restricts promoting TLS for more extensive uses in practice. In order to overcome this problem, new versions of terrestrial laser scanning are needed.

As a state-of-the-art surveying technology, mobile terrestrial laser scanning (MLS) [4] can serve as an alternative solution. Compared to TLS which mostly has only a laser scanner, the configuration of MLS generally incorporates an inertial measurement unit/global navigation satellite system (IMU/GNSS) or an inertial navigation system/global navigation satellite system (INS/GNSS) module, which can supply the real-time attitude/location information for directly georeferencing each laser echo. With the laser scanners and such attitude/location modules integrated together and fixed on the mobile platforms, the resulting MLS systems can continuously get point clouds for featuring the 3D spaces along the routes. Thus, the laborious relocation of the supporting tripods and the re-placement of the reference marks can be largely eliminated. In fact, the concept of MLS has been proposed long before, but its thriving progress mainly spanned the last decade [4]. During that phase, a large number of MLS systems aimed at various applications have been established. The representative MLS systems include the commercial ones like the Riegl VMX-250, StreetMapper, Optech LYNX and Trimble Cougar as summarized in [5] as well as the research-purposed ones like VLMS [6], Roamer [7] and Sensei [8].

Higher measurement efficiency, however, does not mean that MLS performs better than TLS in all of the aspects. MLS also suffers from some limitations in its practical usage. For example, with traveling bumps MLS often tends to present lower stability in the aspect of data georeferencing accuracy. In addition, as MLS often collects data in the way of parallel scan profiles, the sampling resolutions of MLS are easily influenced by the mutative velocities of the moving platforms. These limitations all impact the performance of MLS in object characterization, sometimes even rendering the necessitated spatial information missed. In contrast, for the above-mentioned items, TLS can perform better with higher stability and higher sampling densities. In fact, the strengths of TLS have already been noticed by the MLS community [9].

To synthetically embody the strengths of MLS and TLS, a flexible mapping mode of stop-and-go (sometimes termed as stop-scan-go or stop-go) has been assumed in the MLS mapping field [9,10]. The specific implementation of stop-and-go is to park the moving mapping platform when reaching the target plot and then carry out the mapping of the whole system-surrounding space. Then, the mapping system moves to the next target plot and the same operations are repeated. In fact, the theme of stop-and-go measurement mode has been proposed and applied in a variety of domains, such as for solar energy accumulation in Mars Rover exploration [11] and for simultaneous localization and mapping (SLAM) in robot navigation [12]. Specifically for MLS, data collections in the stop-and-go mapping mode have been utilized as the reference data for, e.g., the calibration of the low-cost mobile mapping system [8] and the assessment of the mapping performance of different MLS systems [13]. Meantime, it is worth mentioning that the stop-and-go mode did not stem only from the process of improving MLS by incorporating the opposite strengths of TLS. It has been earlier recognized by the TLS field suffering from its low measurement efficiency, and some relevant trial works have been conducted (e.g., [14]). Collectively, in both of the fields, the stop-and-go mapping mode, compared to the fully-static (scanning while keeping still) and fully-kinematic (scanning continuously while moving) modes, has been primarily validated as a promising plan for surveying, particularly, special scenarios.

However, the stop-and-go mapping mode emerged in the previous works still as a supplementary way for terrestrial observation in a whole sense. Theoretically, it can be reckoned that its roles have not been fully played. Aimed at this technical gap, this study was dedicated to investigating the potentials of this special mapping mode. Specifically, a relevant literature review was first run individually. Then, the principles of the static and mobile terrestrial laser scanning patterns were analyzed and compared. Next, its power was illustrated by a case study based on the Roamer [7] in the stop-and-go mapping mode. Finally, the strengths of its application were compared and summarized, and the aspects and means involving the enhancement of its application were discussed and suggested, respectively.

## 2. Literature Review

As mentioned above, the stop-and-go mapping mode has been proposed individually in the TLS and MLS fields aimed at their corresponding shortages. In other words, the stop-and-go mapping has been fulfilled based on TLS and MLS systems respectively. Hence, the literature reviews concerning these two fields were implemented separately. The strengths of the stop-and-go mapping mode compared to the fully-static TLS and fully-kinematic MLS mapping modes were also summarized.

### 2.1. TLS-System-Based Works

The so-called TLS-system-based stop-and-go mapping mode is generally implemented based on the traditional TLS systems, which briefly comprise laser scanners and GPS receivers. Even though IMUs sometimes are included, their low-accuracy pose information cannot be relied on. The transition may be involved with little adaptations concerning the components, but their dominant functional modules in hardware are still rooted in the original ones. Namely, TLS systems are just mounted on the mobile platforms for the convenience of their relocations during data collections. However, this minor change proved to release the field-survey engineers from the cumbersome repetitions of carrying-mounting-scanning-dismounting-carrying a laser scanner from one targeted plot to another. After the stop-and-go mapping mode was introduced, the repetition process can be simplified into merely moving-scanning-moving a scanner. The transition from carrying to moving can reduce the *in-situ* labor demands to a large extent, and the cancellation of mounting and dismounting can enhance the efficiency of mapping in a large amount.

Following the above-mentioned scheme plan, some trial works aiming at analysis, optimization and application of the stop-and-go mapping mode have been conducted, particularly in the robot perception and remote sensing fields.

Jones [14] applied the stop-and-go mappings for the extraction of large scale digital terrain models (DTMs) within dangerous mine sites. The assumed TLS system comprised a Riegl Z420i laser scanner and a Trimble R8 GNSS Receiver, and it was mounted to a Caterpillar all-terrain loader. The software used to run the scanner was Riscan Pro. Once all of the scan datasets were registered, the resulted data was manipulated by multi-station adjusted (MSA). MSA ensured that no tilted sets of scan data were provided to users.

Choi *et al.* [15] used the stop-and-go mapping mode to acquire an occupancy grid-based indoor map for mobile robot (mobot) path planning and navigation. The mobot used in the experiment contained a single ultrasonic sensor and a mono-vision system along with a notebook PC, which were mounted on a mobile base.

Jensen *et al.* [16] assumed the stop-and-go data collections in principally addressing the question of generating large-scale 3D models from a set of 2D laser scans. The scanned data was acquired by the Biba-robot using a rotating SICK laser scanner. The robot was moving in a stop-and-go mode through the test site. The initial pose estimation was derived from odometry, which was then fed to the feature-based pose correction and map generation.

De la Puente *et al.* [17] referred to the stop-and-go mapping mode towards mobile robot's enhanced performance in rescue missions. The pioneering 3AT robot Nemo in usage was equipped with a SICK LMS200 laser device mounted on top of a servo pan-tilt (Powercube Wrist 70, Amtec Robotics). A 3D scan was obtained by varying the tilt angle at a constant speed. A data server running on an onboard mini laptop computer sent the synchronized and updated information about odometry, PW70 and laser measurements at client's cyclical requests within a capture procedure.

Heikkilä *et al.* [18] developed a 3D calibration approach for the stop-and-go scanning in highway measurements. The method was not based on any GNSS or inertia-based systems. Four reflectors were installed on the vehicle, and the positions of the reflectors in the project's 3D coordinate system were measured with total station when the vehicle was kept stationary and set into scanning. This provided sufficient information to determine the vehicle's 3D pose (position and orientation). A laser scanner and four active targets (prisms) were installed on the system frame. The frame was ensured to be sufficiently steady and rigid. The system was first calibrated by measuring the prism targets. The laser scanner was controlled remotely by a laptop via TCP/IP using a WLAN connection.

Elhabiby and Teskey [19] summarized the advantages of the stop-and-go laser scanning system by describing the procedure and equipment for data collection and processing. The TLS system assumed for data collection consisted of an automobile with a mounting rack on top. The rack contains three global positioning system (GPS) receivers (at the vehicle corners), and a mounted laser scanner system. There was also a fourth GPS/GNSS receiver, to be place over a known location (for the duration of all scans), to ensure the accuracy of measurement for the three vehicle-mounted GPS receivers. The use of a fourth receiver in this manner allowed for Real Time Kinematic (RTK) processing of GPS/GNSS data [20]. The strength of what was proposed was that GPS/GNSS information was used in lieu of manual alignment of adjacent scans, so that commonly used (and well-understood) ICP algorithm [21,22] was assumed for scan data registration. After the ICP algorithm is performed, GPS/GNSS data were applied to geo-reference the registered scan data. This also saved time in collection and processing the data, because no external geo-referencing information was needed, other than what was collected during stop-and-go mapping.

Pervölz *et al.* [23] used the stop-and-go manner in manipulating robotic systems for tele-exploration. The scan system comprised a standard SICK LMS200 2D laser range finder, which was mounted onto a robot platform Kurt3D. The scan system let rotate the laser scanner continuously around its vertical axis, but accomplished the 3D mapping in a stop-scan-go fashion, therefore acquiring consistent 3D scans as well.

Nüchter *et al.* [12,24,25] assumed the stop-and-go mapping mode in the robot-based SLAM. The used system contained a SICK LMS 291 laser scanner, which was mounted on a mobile robot platform Kurt3D. The 2D laser range finder was attached in the center of rotation to the mount for getting a controlled pitch motion with a standard servo. With the operation modes similar with [23], the scan system let rotate the scanner continuously around its vertical axis, but accomplished the 3D mapping in a stop-scan-go fashion, therefore acquiring consistent 3D scans. A novel scan matching method based on the semantic information was also proposed. In addition to the typical ICP algorithm, the *k-d* trees method [26] has been proposed for performance enhancement.

Chmelina *et al.* [27] designed a system able to most-efficiently (rapidly, automatically) acquire and georeference tunnel wall scans and images in a static (stop-and-go) measuring mode. The developed Orthos Plus prototype consists of a 3D laser scanner (Riegl and Faro scanners are currently supported), a digital camera (e.g., a Nikon D90) and a robotic total station (e.g., Leica TPS1200 series). The three sensors are installed on a light metal frame that can be attached onto a mobile platform (e.g., a hand driven trolley). The platform carries all further needed components, most basically the power supply unit and the control computer with WLAN display.

Pfennigbauer *et al.* [28] used a RIEGL VZ-400 to scan the target area from different scan-positions, simulating a mobile scanning system working in the stop-and-go mode. For the simulation goal, the laser scanner mounted on a tripod and powered by its battery pack was controlled by using a laptop computer via Ethernet TCP/IP link. The internal GPS receiver was used for coarse determination of the different scan-positions. In lieu of employing the information from an INS to match the point clouds, a number of reference targets marked by the reflecting foil were used to determine the relative position and attitude of the scanner for the different scan-positions. With the stop-and-go-simulated multi-scans deployed, the detection of objects concealed by vegetation or camouflage tarps was facilitated.

Carlberg *et al.* [29] used a set of ground-based data scanned in the stop-and-go manner for exploring how to fulfill fast surface reconstruction and segmentation. One of the datasets was acquired using a single 2D laser scanner to obtain terrestrial data in a “stop, scan, and go” way. The scanner is mounted on a stationary platform, rotates about its vertical axis, and incrementally scans the environment until it has obtained a 360°field of view.

### 2.2. MLS-System-Based Works

The MLS-based stop-and-go mappings are facilitated based on the typical MLS systems, commonly with reliable IMU or INS components supplying pose information. It looks like that the mapping is implemented just by parking the carrier supporting the MLS system for one-time measurement of the plot of interest. The minor change is involved in a switch of the system setting from the profile-scan mode to the 360°-scan mode. Compared to the fully-kinematic MLS measurements, the stop-and-go-resulting data tend to perform better in terms of mapping stability and accuracy.

With the performance enhancement of laser scanner in sampling frequency and ranging accuracy, a trend of rotating or pitching the scanner while moving in laser-based mapping, especially in the field of robots for SLAM, once occurred so as to overcome the restrictions of the stop-and-go mapping fashion. This is evidenced by the studies of Wulf *et al.* [30–32] and the achievements of other research groups [33–36]. Some specific examples are presented as follows.

Frueh and Zakhor [35] assumed a vehicle equipped with fast 2D laser scanners and a digital camera while driving at normal speeds on public roads, hence acquiring data continuously rather than in a stop-and-go fashion. That is, the data acquisition was performed in a fast drive-by rather than a stop-and-go fashion, enabling short acquisition times limited only by traffic conditions.

Kümmerle *et al.* [36] used a Mobile Robots Powerbot with a SICK LMS laser range finder mounted on an Amtec wrist unit. The 3D data used for the localization algorithm was acquired by continuously tilting the laser up and down while the robot moved. The maximum translational velocity of the robot during data acquisition was 0.35 m/s. This relatively low speed allows the robot to obtain 3D data that is sufficiently dense to perform scan matching without the need to acquire the scans in a stop-and-go mapping mode.

Then, the implications of the stop-and-go mapping mode were re-emphasized in the MLS domain. Asai *et al.* [10,37] integrated the stop-and-go and continuous scanning of the same rangefinder by registering the overlapped parts of range data for 3D outdoor scene modeling. The system equipped an omni-directional laser rangefinder (Riegl, LMS-Z360), a RTK-GPS (Nikon-Trimble, Log-PakII), and an INS sensor (Tokimec, TISS-5-40). The INS is interlocked with the RTK-GPS in order to correct the cumulative error by measuring the direction of movement derived from GPS data during movement. This hybrid sensor module facilitated acquiring the position and orientation with high accuracy by compensating the low measurement rate of the RTK-GPS and the cumulative error of the INS sensor.

Hyyppä *et al.* [9] used MLS data collections in the stop-and-go mode instead of the conventional TLS mapping as the reference for urban tree change detection. The advantage is that a TLS system can be reduced in the campaigns of MLS mapping. The stop-and-go mobile mapping data collected by the Roamer system [7] was used as the reference data for calibration of the low-cost mobile mapping system [8]. The stop-and-go mobile mapping data has also been used for tree height monitoring [13], biomass estimation at individual tree level [38], tree crown attributes investigation [39], snow cover profiling [40] and culvert detection [41]. So far, the stop-and-go mode in the MLS-based mappings is mainly applied for providing the high-accuracy reference data.

Colombo *et al.* [42,43] developed the software module with the stop-and-go mapping solution for rapid mobile surveys with pre-surveyed way-points, and this module was aimed at fulfilling large-area, sub-decimeter positioning for airborne LiDAR surveys.

## 3. Principles Analysis

The literature review primarily presented the promising roles of the stop-and-go mode in terrestrial laser mapping. However, different variants of terrestrial laser scanning show different features, and the generic principles of TLS-system-based and MLS-system-based stop-and-go mapping modes need to be analyzed respectively. Then, with their individual strengths absorbed, more efficient stop-and-go mapping modes in mobile terrestrial laser scanning can be designed.

### 3.1. TLS-System-Based Mode

**Figure 1.**
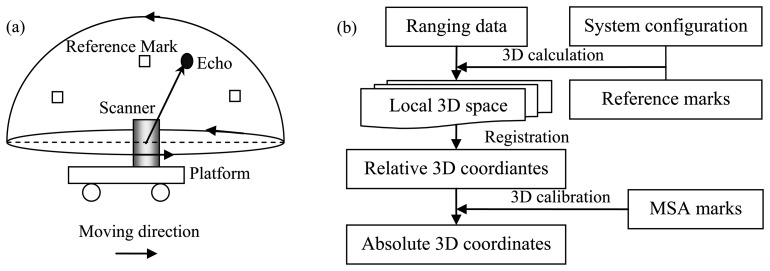
(**a**) The schematic diagram of TLS-system-based stop-and-go mapping principle. (**b**) The workflow of georeferencing TLS-system-collected point clouds.

The typical schematic principle of the TLS-system-based stop-and-go mapping mode is illustrated in Figure 1(a). The components involve one laser scanner and some reference marks fixed to the TLS system frame, and the relative locations of the marks are measured in advance using, e.g., GPS. When the platform stops at the targeted plot, the laser scanner emits and receives laser pulses within its preset scan profile and simultaneously rotates continuously around its axis, therefore acquiring consistent 3D scans. The workflow of georeferencing the TLS-system-collected point cloud is shown in Figure 1(b).

The first step of georeferencing is to convert the 1D ranging data recorded by the laser scanner into 3D coordinates. The 3D calculation procedure is based on the projection model derived from the configuration of the TLS system and the reference marks. The specific transformation matrix can refer to [19] and [44]. However, the resulted data can only characterize each local 3D plot space. In order to represent the whole site for mapping, registration is necessitated. A number of registration algorithms have been attempted for acquiring a consistent 3D representation of the whole target space. The related 3D registration methods such as ICP can refer to [21] and [22]. Further, in order to automate the ICP algorithms, a neighborhood search based technique proposed by Bae and Lichti [45] and a method by integrating image data to supplement scan data proposed by Dold and Brenner [46] were attempted. Yet, the resulted data are merely a representation of the mapped region with relative 3D coordinates. For practical applications, the absolute 3D coordinates need to be acquired. This is implemented by MSA, which can ensure that no tilted sets of scan data are provided to users. The 3D calibration is fulfilled based on the MSA marks accurately measured by such as RTK-GPS.

### 3.2. MLS-System-Based Mode

**Figure 2.**
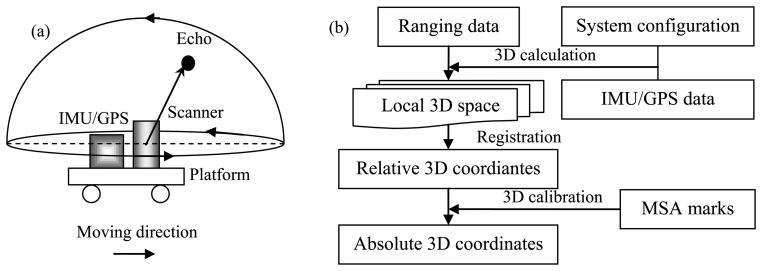
(**a**) The schematic diagram of MLS-system-based stop-and-go mapping principle. (**b**) The workflow of georeferencing MLS-system-collected point clouds.

The typical schematic principle of the MLS-system-based stop-and-go mapping mode is illustrated in Figure 2(a). The system comprises a laser scanner and a set of IMU/GPS sensors, which are fixed on the frame with the pre-determined relative locations. When the platform stops at the targeted plot, the laser scanner emits and receives laser pulses within its preset scan profile and simultaneously rotates continuously around its axis as TLS, acquiring consistent 3D scans in the end. The workflow of georeferencing the MLS-system-collected point cloud is shown in Figure 2(b).

The process of georeferencing MLS-system-collected data is similar with the TLS-related one. The differences lie mainly on the IMU/GPS module, which can directly supply the pose information of the whole system. This change makes the setup of the mapping system simpler. This adding also facilitates a direct 3D projection, sometimes even with the absolute 3D coordinates output if the accuracy of the IMU/GPS is high enough. The specific transformation matrix can refer to [7] and [37]. In addition, in order to represent the whole region of interest, registration is necessitated as in the scenario of TLS-system-based mapping. The related 3D registration methods such as ICP [21,22] are also available for the MLS-system-collected data registration. At the same time, the algorithms aimed at the special features of MLS have also been put forward, such as by Rieger *et al.* [47]. Finally, the MSA-based calibration also needs to be manipulated to render absolute 3D coordinates with high accuracy.

### 3.3. Step-forwards in Principle

From principle analysis, the specific schematic plans of the stop-and-go mapping mode based on TLS and MLS systems can be generalized as listed in Table 1. The already-implemented modes refer to three types, *i.e.*, TLS-system-based 3D-scan when the platform is kept static (termed as TLS-based), MLS-system-based 3D-scan when the platform is kept static, and MLS-system-based 3D-scan but with sampling density adapted higher when the platform is kept static. As both of the MLS-system-based modes for most of the existing MLS systems now still need some manual operations and cannot transit directly from the fully-kinematic mapping mode, they are termed as MLS-based (M-1) and MLS-based (M-2) respectively. Now, the relevant studies are focusing on fulfilling MLS-based (M-2) mode.

It is worth mentioning that from the perspective of efficiency, the two existing plans of MLS-based stop-and-go mapping up to manual switch-on/off manipulations are still far from enough. If automatic control is introduced, the stop-and-go mapping can be continuously implemented along with the fully-kinematic mapping. Accordingly, two different specific schematic plans for the stop-and-go mapping mode can be figured out, as listed in Table 1. In these two schematic plans, MLS systems run profile-scans when moving and then run 3D-scans when parking. Their only difference is to maintain or raise their sampling densities in the “stop” pattern compared to the “go” pattern. Correspondingly, the two mapping modes in automatic way are termed as MLS-based (A-3) and MLS-based (A-4).

## 4. Case Study

The MLS system assumed for case study is the Roamer [7]. It comprises a FARO LS 880HE80 laser scanner for 3D mapping, with its spatial trajectory derived by the NovAtel Synchronized Position Attitude Navigation (SPAN) technology. In addition, Roamer can perform panoramic scans when the mobile platform is held static (*i.e.*, stop-and-go), as FARO LS 880HE80 originally was designed to be a terrestrial laser scanner with a field-of-view 320° × 360°.

The data for a study of built environment representation was collected at the Espoonlahti district in Southern Finland, a typical urban street environment, and the same data was used here. The Roamer-based fully-kinematic MLS campaign was deployed on 10 June 2009. The Roamer-based stop-and-go mapping was conducted on 7 May 2009, but its scan resolution was set to 1/8 of the value specified in its fully-kinematic collection. That is, its maximum sampling rate can reach 960,000 hits per second.

**Figure 3.**
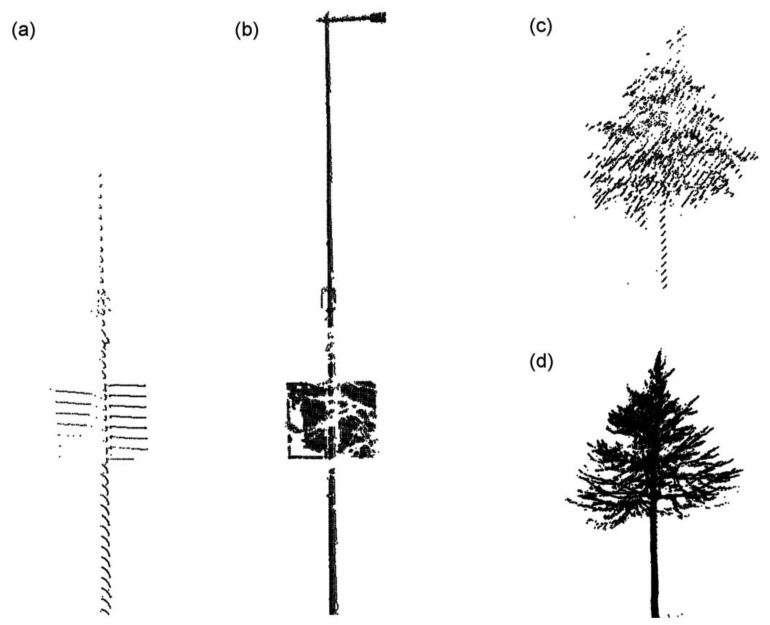
(**a**) Illustration of lighting pole reconstruction based on the fully-kinematic MLS data; (**b**) The same lighting pole reconstruction based on the stop-and-go data mapped by the same MLS system; (**c**) Illustration of tree reconstruction based on the fully-kinematic MLS data; (**d**) The same tree reconstruction based on the stop-and-go data mapped by the same MLS system.

The advantages of the stop-and-go mapping mode can be illustrated by the case study in Figure 3. The cases involve the reconstructions of a lighting pole and a tree, which maintain almost the equal distances to the laser scanner in the two mapping modes. From Figure 3, it can be clearly learnt that the lighting pole reconstructed from the stop-and-go data is more complete than the opposite one from the fully-kinematic data. For example, the advertisement board is relatively-completely represented by the stop-and-go data, while the lighting pole branch is even missed in the fully-kinematic MLS surveying. For the same tree, its details like thin branches can be reflected by the stop-and-go mapping data better than the fully-kinematic Roamer MLS data, although in the latter scenario laser pulses were somehow impacted by the growing leaves. The two cases can intuitively validate the strength of the stop-and-go mapping mode.

## 5. Discussions and Suggestions

### 5.1. Summary of the Advantages—Compared to Fully-static TLS

The principal advantage of the stop-and-go MLS/TLS compared to the fully-static TLS mapping mode is efficiency. This is also the strongest motive urging the researchers to develop the stop-and-go mode in the TLS field. The efficiency is reflected in multiple aspects as follows.

First, the stop-and-go MLS/TLS can save time in the measurement campaigns. As reviewed in the Section 2.1, the stop-and-go MLS/TLS can replace the traditional operations of carrying-mounting-scanning-dismounting-carrying with moving-scanning-moving. Specifically, it is only required to drive between subsequent scan sites, rather than move the equipment from one site to another. Moreover, the repeated manual manipulations of setting-up and taking-down the laser scanner from the tripod in each mapping site are omitted. All of these operation simplifications can save a lot of time.

Second, the stop-and-go MLS/TLS mode can save expenditure in the field inventories. As the TLS systems have not received many adaptations, it can be reckoned that the cost in terms of hardware is constant. The reduction of expenditure is mainly up to the decrease in the number of survey engineers. In traditional fully-static TLS-based mapping, more than one crew is required, because sometimes scan equipments are so heavy to carry. Instead, the stop-and-go MLS/TLS mapping is vehicle-based and only requires a single operator in all cases. The reduction of labor cost can save a lot of money.

Third, the stop-and-go MLS/TLS can add the richness of samples under the same measurement conditions. Compared to time and money saving, sample increasing in some cases is more important for training the information retrieval models. With the high mobility enabled by vehicles, the stop-and-go MLS/TLS can cover larger areas. Larger areas mean more diverse samples with the representative significances. More samples tend to mean more accurate and reliable models for information retrieval. In fact, sample collection is a conventional issue in the fields of earth observation and remote sensing, and thus, the stop-and-go MLS/TLS mode is particularly useful.

### 5.2. Summary of the Advantages—Compared to Fully-Kinematic MLS

The primary advantage of the stop-and-go MLS in contrast to the fully-kinematic MLS mapping mode is stability. This is also the reason for the researchers to re-highlight the stop-and-go mode in the MLS field. The stability is characterized by many factors as follows.

First, the stop-and-go MLS mapping can maintain accuracy in the survey campaigns. Although the fully-kinematic MLS systems can theoretically achieve similar accuracies as the stop-and-go mapping mode does, the practices are not like so. The reason is that the fully-kinematic MLS systems are often driven on uneven terrains. The bumps of the platform may impact the performance of IMU/GPS unit. In contrast, the stop-and-go mapping mode is much more suited to producing consistent high quality and accurate scan data.

Second, the stop-and-go MLS mapping can enhance the completeness in target representation. As the fully-kinematic MLS systems generally measure targets in the mode of parallel scan profiles, the details of targets between two adjacent scan profiles are often missed. This is intractable if the vehicle moves with varying speeds. But for the stop-and-go mapping mode, its scanning rate is constant. Thus, the targets can be better characterized by the laser echoes with stable angles, especially when the scan frequency is raised, e.g., in the scenario of MLS-based (M-2). Higher sampling density tends to render higher completeness of target representation.

In addition, an important merit of the stop-and-go TLS compared to the fully-kinematic MLS is cost. The stop-and-go TLS systems are typically in lower expenditure than the fully-kinematic MLS systems, because the latter ones generally incorporate the IMU/GNSS modules. The pose/position modules able of facilitating the major functions of MLS are so expensive, and the prices of MLS systems are often tripled opposite to TLS. At the same time, the stop-and-go TLS systems are almost as efficient as the fully-kinematic MLS systems in gathering data. Thus, the low-price stop-and-go TLS systems are very attracting to the research groups with funding in shortage.

### 5.3. Suggestions

From the literature review and the case study, it can be derived that stop-and-go as an implementing way of sensor manipulation is a promising mapping mode for terrestrial laser scanning, and further, sensor manipulation is as important as sensor development in a generic sense. Stop-and-go performs with many strengths, e.g., with better efficiency than the fully-static TLS and with better stability than the fully-kinematic MLS. However, its current studies only reach the MLS-based (M-2) stage, which still demands the engineers to manually switch on/off or tune the laser scanner when arriving at the test plot. At the same time, the affiliated mechanism analysis and method development for its performance improvement are still far from enough. Thus, the stop-and-go mapping mode in the field of terrestrial laser scanning is yet to be explored.

From the perspective of function enhancement, the particular features embedded in the stop-and-go data collection need to be explored. For example, does it have any differences with the conventional fully-static TLS in terms of measurement mechanism? Then, aimed at such features, appropriate and powerful algorithms for information extraction need to be developed. For example, how to get balance between coping with high sampling density and ensuring data-processing efficiency?

From the perspective of system coordination, more measures about automatic controlling need to be introduced into the stop-and-go mapping mode. For example, how to maintain continuous scanning from the “stop” mode to the “go” mode? Further, for this case, the software modules concerning data-processing algorithms can robustly handle the datasets of different mapping modes, even in the pattern of on-line data processing when the vehicle moves.

Specifically, the above-mentioned works can be carried out by implementing the mapping modes of MLS-based (A-3) and MLS-based (A-4). The advantages of utilizing the mapping modes A-3 and A-4 in Table 1 are obvious. With A-3 and A-4, the target areas, where improved mapping quality (accuracy, density, stability) is required, can be automatically measured with 3D-scan mode. The vehicle stops, until then the system measures in the fully-kinematic mode. After the 3D scan, the vehicle continues. The 3D scan provides a rigid geometry, and other scan-profile data can also be fitted to that, *i.e.* the 3D scan data are the reference data used for georeferencing of the fully-kinematic data.

Overall, the next-step research plans potential for improving the stop-and-go mapping mode can be suggested. That is, more algorithms appropriate for the features of the stop-and-go mapping mode need to be developed, and the two enhanced stages (MLS-based (A-3) and MLS-based (A-4)) need to be fulfilled in the field of mobile terrestrial laser scanning in the future.

## Figures and Tables

**Table 1.** The specific schematic plans of the stop-and-go mapping mode based on TLS and MLS systems.

	**Go**	**Stop**	**Go**	**Schematic term**
TLS	-	3D-scan	-	TLS-based

	-	3D-scan	-	MLS-based (M-1)
MLS	-	3D-scan (higher sampling density)	-	MLS-based (M-2)
	Profile-scan	3D-scan	Profile-scan	MLS-based (A-3)
	Profile-scan	3D-scan (higher sampling density)	Profile-scan	MLS-based (A-4)
